# Evaluating short-term and survival outcomes of natural orifice specimen extraction surgery for colorectal cancer: A single-centre retrospective study

**DOI:** 10.3389/fsurg.2023.1078316

**Published:** 2023-02-23

**Authors:** Fuqiang Zhao, Wei Zhao, Tixian Xiao, Zhijie Wang, Fei Huang, Wei Xing, Qian Liu

**Affiliations:** ^1^ Department of Colorectal Surgery, National Cancer Center/National Clinical Research Center for Cancer/Cancer Hospital, Chinese Academy of Medical Sciences and Peking Union Medical College, Beijing, China; ^2^ Department of General Surgery, Hebei Province Hospital of Chinese Medicine, Affiliated Hospital of Hebei University of Chinese Medicine, Shijiazhuang, China

**Keywords:** natural orifice specimen extraction surgery (NOSES), laparoscopic surgery, colorectal cancer, survival, short-term outcomes

## Abstract

**Background:**

Natural orifice specimen extraction surgery (NOSES) has been confirmed as an alternative approach without auxiliary incisions. The purpose of this study was to investigate the short-term and survival outcomes of NOSES versus conventional laparoscopic surgery (LAP) in treatment of sigmoid and high rectal cancer.

**Method:**

The retrospective study was conducted at single centers between January 2017 to December 2021. Relevant data included clinical demographics, pathological features, operative parameters, postoperative complications and survival outcomes were collected and analyzed. All procedures were performed using either a NOSES or a conventional LAP approach. Propensity score matching (PSM) was conducted to balance clinical and pathological features between the two groups.

**Results:**

After PSM, a total of 288 patients were eventually included in this study, 144 in each group. Patients in the NOSES group experienced faster recovery of gastrointestinal function (2.6 ± 0.8 vs. 3.6 ± 0.9 day, *P *= 0.037), less pain and less analgesia required (12.5% vs. 33.3%, *P *< 0.001). In addition, the incidence of surgical site infection in the LAP group was significantly higher than that in the NOSES group (12.5% vs. 4.2%, *P *= 0.011), especially incision-related complications (8.3% vs. 2.1%, *P *= 0.017). After a median follow-up of 32 (range, 3–75) months, the two groups had similar 3-year overall survival rates (88.4% vs. 88.6%; *P *= 0.850) and disease-free survival rates (82.9% vs. 77.2%; *P *= 0.494).

**Conclusion:**

The transrectal NOSES procedure is a well-established strategy with advantages in reducing postoperative pain, faster recovery of gastrointestinal function, and less incision-related complications. In addition, the long-term survival is similar between NOSES and conventional laparoscopic surgery.

## Introduction

In the past 20 years, laparoscopic surgery (LAP) has been widely used in the treatment of colorectal cancer (CRC). Many studies have clarified that compared with open surgery, LAP has the advantages of less trauma and faster recovery while ensuring the same therapeutic effect, which is an important milestone in the development of modern surgery (1, 2). In traditional LAP, tumor specimens were removed through a small abdominal incision with a length of about 6–8 cm, and then an anvil head attached to circular stapling device was inserted to complete the reconstruction of digestive tract. To some extent, the minimally invasive advantages of LAP were offset, and incision-related complications were increased, affecting the immune function of the body.

Natural orifice specimen extraction surgery (NOSES) can be easily combined with existing LAP skill without the need for additional specialized equipment. In the past few years, NOSES have been successfully promoted and developed in colorectal cancer treatment, and transrectal and transvaginal approaches are common natural method for extraction of specimens (3–8). However, NOSES is currently exploratory and there is no high-grade evidence of evidence for its clinical outcomes, especially the long-term survival outcomes. Therefore, we conducted a single centre, case-matched analysis to demonstrate the safety and feasibility of NOSES in treatment for colorectal cancer by comparing the short-term and survival outcomes with those of conventional LAP.

## Patient and methods

**Figure 1:**
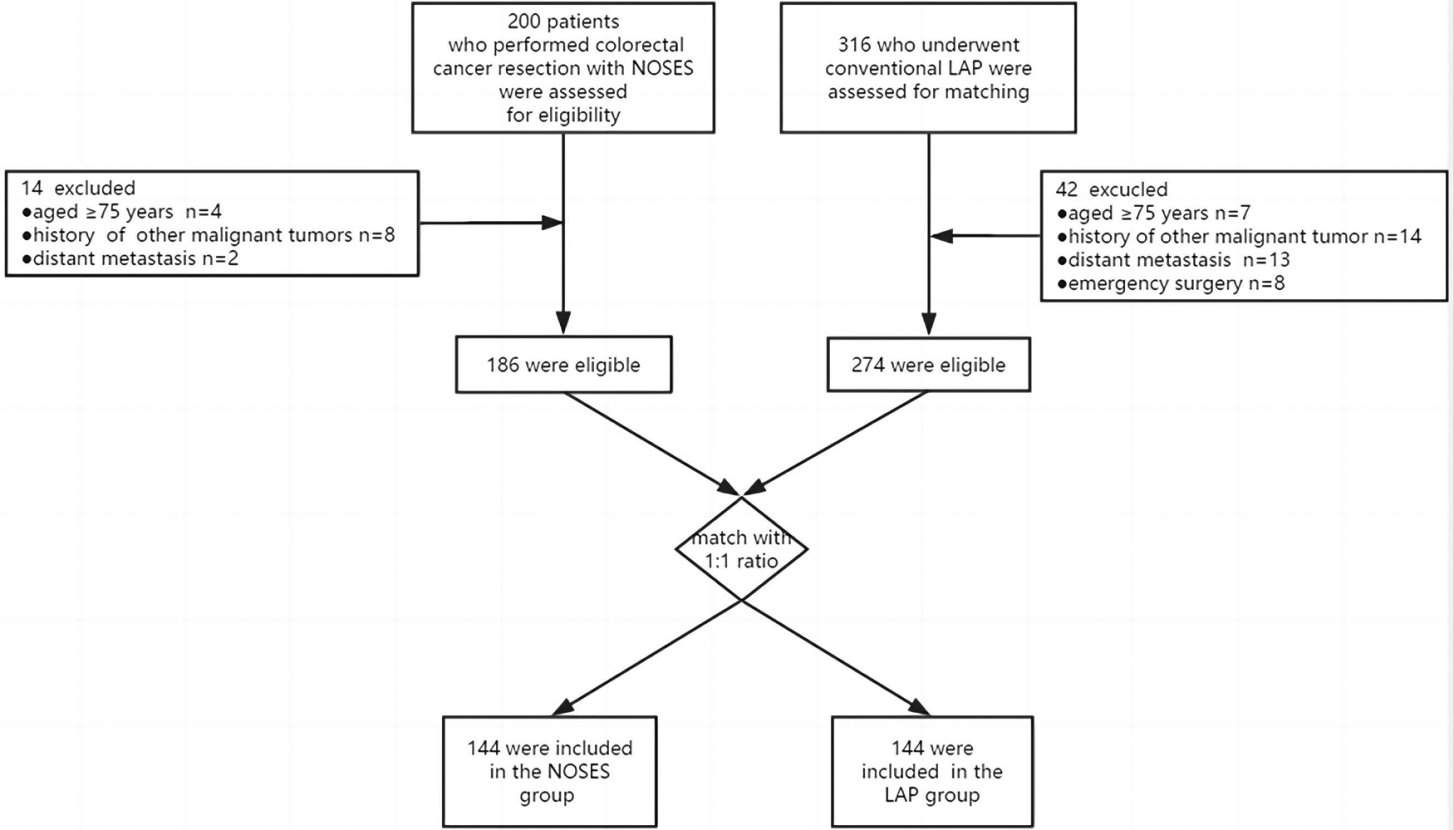
Patient selection flowchart.

From January 2017 to December 2021, patients underwent laparoscopic radical resection in treatment for upper rectal cancer and sigmoid colon cancer at the National Cancer Center were reviewed. Patients who underwent laparoscopic surgery with natural orifice extraction were assigned to NOSES group while patients who performed conventional laparoscopic surgery with abdominal auxiliary incision were assigned to LAP group. The inclusion criteria were as follows: (1) aged between 18 and 75 years; (2) cT stage 1–3 (3) without distant metastasis. The exclusion criteria were: (1) complicated with other malignant tumors (2) emergency surgery for acute intestinal obstruction, perforation or bleeding. Finally, 186 patients who underwent NOSES and 274 who underwent LAP were enrolled. Propensity score matching (PSM) was used to balance the baseline data between the two groups. Propensity scores were matched 1:1 based on age, gender, body mass index (BMI), American Society of Anaesthesiologists (ASA) score, preoperative chemotherapy, tumor location, tumor differentiation, T stage, N stage, and tumor size. Finally, 144 patients were assigned to the NOSES group while 144 patients were assigned to the LAP group (Figure 1).

Preoperative assessment for all patients included laboratory examination, colonoscopy with biopsy, abdominal CT scan and pelvic magnetic resonance imaging. Tumor staging was evaluated according to the American Joint Committee on Cancer (AJCC, eighth edition) staging system. Patients with clinical stage II and III received preoperative chemoradiotherapy followed by surgery 6 weeks later. All patients received mechanical bowel preparation before surgery, and intravenous antibiotic profilaxis were administered during perioperative period. Postoperatively, patient-controlled analgesia (PCA) was administered to all patients for pain management, and additional nonsteroidal and opioid analgesics were administered intravenously as required. Pain scores were assessed once daily with a validated visual analogue scale (VAS), ranging from 0 to 10, with 0 representing no pain and 10 representing the worst conceivable pain. All patients signed the written informed consent and complied with the Declaration of Helsinki. This retrospective study was approved by the Institutional Review Board Committee of the Cancer Hospital at the Chinese Academy of Medical Sciences (No. 18-015/1617).

### Surgical procedure

The modified lithotomy position was taken, and all patients adopted the five trocar position with a pneumoperitoneum of 15 mmHg. One trocar was placed supraumbilical for the camera, while two trocars were placed on the right and left quadrant, respectively. After careful exploration, the patient was placed in the Trendelenburg position. The standard surgical technique was performed both in NOSES group and LAP group including separation and high ligation of the inferior mesenteric vessel, mobilization of the bowel, and dissection of the lymph nodes and and division of the distal rectum. Then the specimen extraction approach was different in two group. After the operation, both groups of patients will have 2 drainage tubes in the pelvic cavity, which are usually removed 5–7 days after the operation.

For LAP group, an auxiliary abdominal incision 6–8 cm in length was made for specimen extraction. Then, the anastomosis was performed by a double-stapling technique under the direct visual observation.

**Figure 2:**
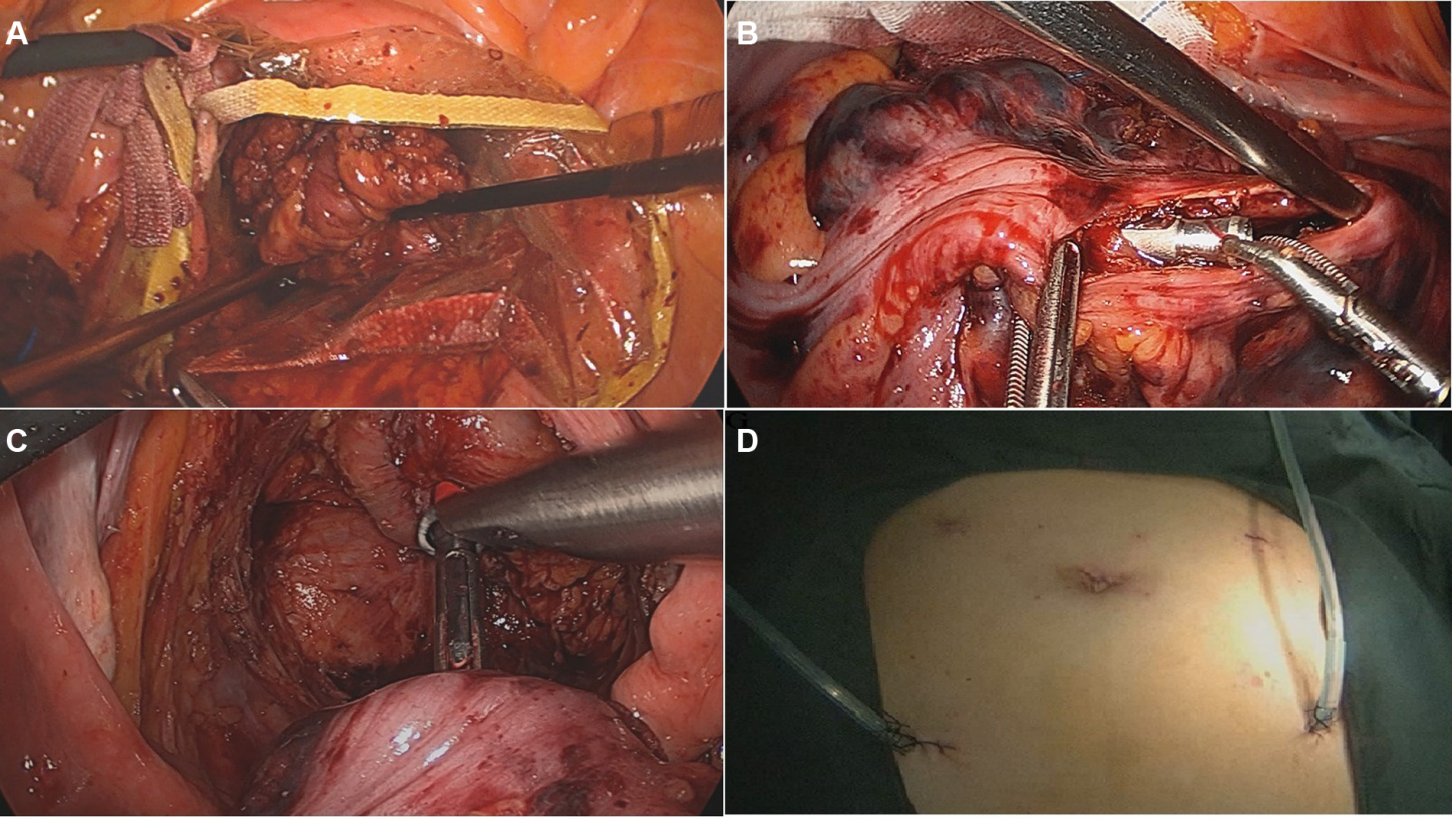
The surgical procedure of NOSES. (**A**) The specimen was pulled out through the disposable sterile protective cover; (**B**) the anvil head was inserted into the colon lumen through the incision; (**C**) end-to-end colorectal anastomosis was performed with the use of a circular stapling device; (**D**) no auxiliary incision in the abdomen after procedure.

For NOSES group, after mobilization of the rectum and left colon, the distal rectum was transected below the tumor with a linear stapler. An incision was generated below the staple line of the rectal stump and a sterile plastic sleeve was placed into the abdominal cavity through the anus and rectal stump. Next, a long Babcock grasper was brought through the anus, and the specimen was extracted through the plastic sleeve (Figure 2A). Then, an anvil head attached to circular stapling device was inserted into the abdominal cavity, and a longitudinal incision approximately 2 cm was made on proximal colon wall to insert the anvil head (Figure 2B). Subsequently, the proximal colon was transected in close proximity to the upper pole of the incision using a linear stapler. Next, the rectum stump was transected with a linear stapling device. Finally, end-to-end colorectal anastomosis was performed with the use of a circular stapling device (Figure 2C). After the procedure, there is no auxiliary incision in the abdomen (Figure 2D).

### Follow-up

According to the guidelines of the NCCN, enrolled patients with T3/T4 or N+ received adjuvant therapy after operation if they did not receive preoperative treatment. All patients were scheduled to receive follow-up through outpatient visits every 6 months in the first 3 years. Physical exam, blood test (including CEA and CA19-9), and CT scans (chest, abdomen and pelvic) were completed at each follow-up. Three years after surgery, the patients were followed up every 12 months by outpatient visits or telephone, the deadline for the follow-up was December 1, 2021. The survival endpoints of present study were 3-year overall survival (OS) and disease-free survival (DFS).

### Statistical analysis

Statistical data were processed using the SPSS software version 24.0 for Windows (IBM Crop, Armonk, NY, United States). Quantitative data are expressed as the mean ± standard deviation and categorical data are expressed as percentages. The differences in classification or quantitative variables were analyzed using *t* test or chi-square test, respectively. DFS and OS were calculated using the Kaplan-Meier method and the differences were compared by a log-rank test. A *P* value of <0.05 was considered statistically significant.

## Results

### Baseline characteristic

Consecutive patients with sigmoid colon or upper rectal cancer undergoing laparoscopic radical resection were enrolled between January 2017 to December 2021. A total of 460 patients from our center were included in this study, including 274 in the LA group and 186 in the NOSES group.

The baseline characteristic were presented in Table 1. Before PSM, NOSES group and LAP group were unevenly distributed in baseline characteristics such as age (58.7 ± 10.3 vs. 61.9 ± 11.4 years, *P *< 0.001), BMI (23.0 ± 28 vs. 24.2 ± 3.3 kg/m^2^, *P *= 0.042), ASA score, preoperative chemotherapy (*P *< 0.001), T stages (*P *< 0.001), N stage (*P *= 0.001), and tumor size (3.5 ± 1.3 vs. 4.8 ± 1.6 cm, *P *< 0.001). After PSM, the clinical variables, including age, gender, BMI, ASA category, preoperative chemoradiotherapy, tumour location, tumor differentiation, T stage, N stage and tumor size, were well balanced between the two groups (all *P* < 0.05).

**Table 1:** Clinical and pathologic characteristics before and after PSM.

Variables	Original cohort			Matched cohort		
	LAP (*n* = 274)	NOSES (*n* = 186)	*P*	LAP (*n* = 144)	NOSES (*n* = 144)	*P*
Age (years, mean ± SD)	61.9 ± 11.4	58.7 ± 10.3	<0.001	59.9. ± 11.8	58.9 ± 10.2	0.422
Gender			0.476			0.720
Male	141 (51.5)	102 (54.8)		85 (59.0)	82 (56.9)	
Female	133 (48.5)	84 (45.2)		59 (41.0)	62 (43.1)	
BMI (kg/m^2^, mean ± SD)	24.2 ± 3.3	23.0 ± 2.8	0.042	23.4 ± 3.1	23.3 ± 2.8	0.855
ASA score			0.011			0.294
I–II	213 (77.7)	162 (87.1)		113 (78.5)	120 (83.3)	
III–IV	61 (22.3)	24 (22.9)		31 (21.5)	24 (16.7)	
Preoperative chemotherapy			<0.001			0.199
Yes	76 (27.7)	23 (12.4)		32 (22.2)	23 (16.0)	
No	298 (72.3)	233 (87.6)		224 (77.8)	233 (84.0)	
Tumor location			0.698			0.811
Upper rectum	163 (59.5)	114 (61.3)		84 (58.3)	86 (59.8)	
Sigmoid colon	111 (40.5)	72 (38.7)		60 (41.7)	58 (40.2)	
Tumor differentiation			0.271			0.924
Poor	46 (16.8)	24 (12.9)		24 (16.7)	22 (15.3)	
Median	200 (73.0)	148 (79.6)		102 (70.8)	105 (72.9)	
High	28 (10.2)	14 (7.5)		18 (12.5)	17 (11.8)	
pT stage			0.004			0.437
T1–T2	83 (30.3)	81 (43.5)		39 (27.1)	45 (31.3)	
T3–T4	191 (69.7)	105 (56.5)		105 (72.9)	99 (68.7)	
pN stage			0.001			0.341
N0	154 (56.2)	132 (71.0)		78 (54.2)	86 (59.7)	
N1/N2	120 (43.8)	54 (29.0)		66 (45.8)	58 (40.3)	
Tumor size (cm, mean ± SD)	4.8 ± 1.6	3.5 ± 1.3	<0.001	3.8 ± 1.4	3.6 ± 1.3	0.553

BMI, body mass index; ASA, American Society of Anesthesiologists; PSM, propensity score matching.

### Short-term outcomes

The surgical details and postoperative outcomes are listed in Table 2. The mean operative time (NOSES 145.9 ± 42.1 min vs. LAP 135.8 ± 43.7 min; *P* = 0.153) and estimated blood loss were essentially identical (NOSES 39.6 ± 4.3 ml vs. LAP 40.5 ± 29.3 ml; *P* = 0.832). The mean numbers of retrieved lymph nodes were 20.8 ± 2.3 and 21.1 ± 8.0 in the NOSES group and LAP group, respectively (*P *= 0.577). Patients in the NOSES group suffered significantly less pain than those in the LAP group on day 1–3 (*P* < 0.05) and fewer patients in NOSES group required analgesia (12.5% vs. 33.3%, *P *< 0.001). Additionally, time to recovery of gastrointestinal functions was shorter in the NOSES group than in the LAP group (2.6 ± 0.8 vs. 3.6 ± 0.9 day, *P *= 0.037). With regard to postoperative complications, 13 (9.0%) patients developed postoperative complications in NOSES group and 18 (12.5%) patients in LAP group (*P *= 0.342). However, the incidence of surgical site infection in the LAP group was significantly higher than that in the NOSES group (12.5% vs. 4.2%, *P *= 0.011), especially incision-related complications (8.3% vs. 2.1%, *P *= 0.017). There was one surgery-related deaths occurred in the NOSES group within 30 days after surgery while the mortality rates between the NOSES group and LAP group were not significantly different (0% vs. 0.7%, *P *= 1.000).

**Table 2:** The perioperative outcome of patients in the NOSES group and LAP group.

Variables	LAP (*n* = 144)	NOSES (*n* = 144)	*P*
Operation time (min, mean ± SD)	135.8 ± 43.7	145.9 ± 42.1	0.153
Estimated blood loss (ml, mean ± SD)	40.5 ± 29.3	39.6 ± 34.3	0.832
Lymph node harvest (mean ± SD)	21.1 ± 8.0	20.8 ± 8.3	0.577
Additional analgesia required [*n* (%)]	18 (12.5)	48 (33.3)	<0.001
Time to recovery of gastrointestinal function (day, mean ± SD)	3.6 ± 0.9	2.6 ± 0.8	0.037
Postoperative hospital stay (day, mean ± SD)	8.2 ± 2.4	7.8 ± 2.2	0.322
Postoperative complication [*n* (%)]	18 (12.5)	13 (9.0)	0.342
Anastomosis leakage	3 (2.1)	6 (4.2)	0.310
Anastomotic bleeding	2 (1.4)	3 (2.1)	1.000
Ileus	4 (2.8)	3 (2.1)	1.000
Pneumonia	2 (1.4)	2 (1.4)	1.000
Surgical site infection	18 (12.5)	6 (4.2)	0.011
Incision-related complications	12 (8.3)	3 (2.1)	0.017
Pelvic abscess	4 (2.8)	3 (2.11)	1.000
Reoperation [*n* (%)]	3 (2.1)	2 (1.4)	0.680
Mortality [*n* (%)]	0 (0)	1 (0.7)	1.000
**Pain score (VAS, mean ± SD)**			
POD1	3.4 ± 1.2	2.1 ± 1.3	<0.001
POD2	3.0 ± 1.1	2.0 ± 0.9	<0.001
POD3	2.1 ± 1.1	1.5 ± 0.9	0.011
POD4	1.6 ± 0.8	1.4 ± 0.8	0.622
POD5	1.4 ± 0.7	1.3 ± 0.8	0.763

VAS, visual analogue scale; POD, postoperative day.

### Survival outcomes

**Figure 3:**
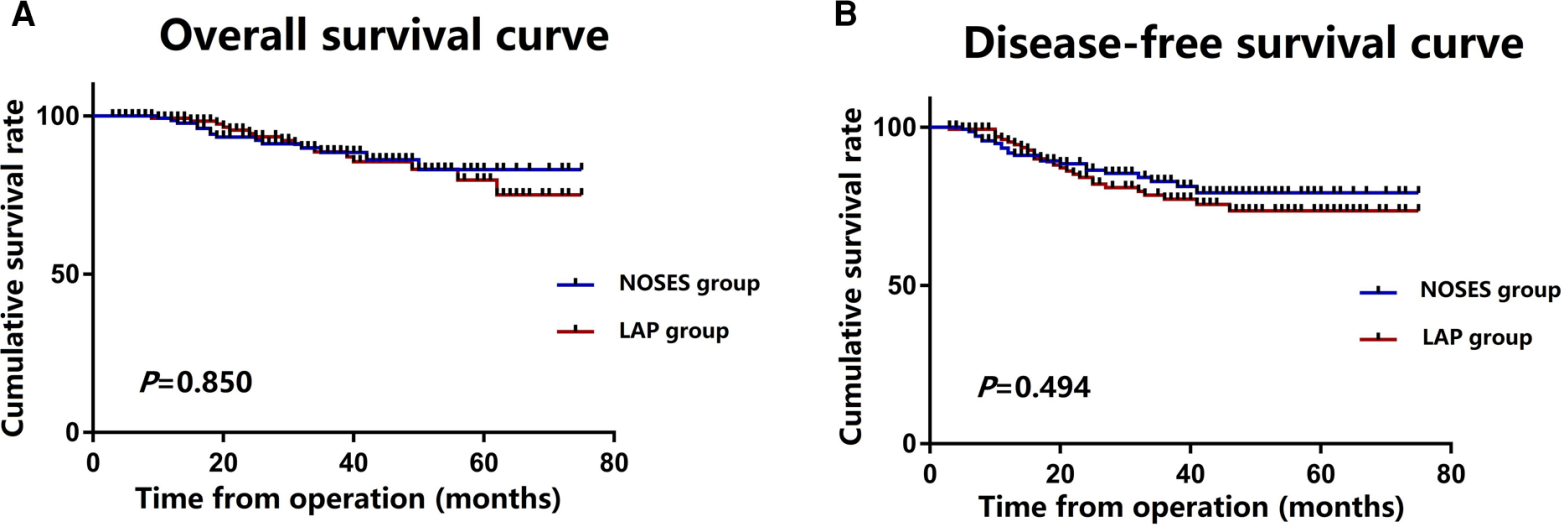
Survival outcomes for the NOSES and LAP groups; (**A**) overall survival in the NOSES and LAP groups; (**B**) disease-free survival in the NOSES and LAP groups.

The median follow-up period was 32 (range, 3–75) months. During the follow-up period, 26 of the 288 patients died, and 42 patients had local recurrence or distant metastasis. There was no significant difference in tumour recurrence between the NOSES group and the LAP group. 5 patients developed local recurrence and 17 patients developed distant recurrence in the NOSES group after a median follow-up of 31 (range, 3–75) months. 6 patient developed local recurrence and 14 patients developed distant recurrence in the LAP group after a median follow-up of 33 (range, 4–75) months. The Kaplan curves showed that the NOSES group had similar OS (*P* = 0.850) and DFS (*P* = 0.494) compared with the LAP group (Figures 3A,B). The 1, 3-year OS rate in the NOSES group were 98.5% and 88.4%, respectively, and those in the LAP group were 93.3% and 82.9%, respectively. The 1, 3-year DFS rate in the NOSES group were 99.2% and 88.6%, respectively, and those in the LAP group were 95.3% and 77.2%, respectively (Table 3).

**Table 3:** Overall survival and disease-free survival of patients in NOSES group and LAP group.

	*N*	Overall survival		Disease-free survival	
		1-year	3-year	1-year	3-year
NOSES	144	98.5%	88.4%	93.3%	82.9%
LAP	144	99.2%	88.6%	95.3%	77.2%

## Discussion

Currently, surgery is the only radical treatment for CRC, however, it will inevitably bring trauma, pain, scar and other side effects. With the continuous development of laparoscopic technology, NOSES surgery can avoid abdominal auxiliary incisions, minimize pain, consistent with minimally invasive principles (9–11). This single center-retrospective study compared the short-term and survival outcomes between patients who performed NOSES and the patients who performed conventional LAP using a case-matched design.

The characteristic of NOSES is that the specimen is extracted from the natural orifice, avoiding the auxiliary abdominal incision of 6–8 cm. It has been reported that the incidence of incision infection in traditional LAP surgery is about 5%–10% (12), while the incidence of incision hernia is between 15% and 20% (13). In addition, incision infection is an independent risk factor for long-term survival in patients with CRC (14). Our study found that the incidence of incision-related complications in the LAP group was significantly higher than that in the NOSES group (8.2% vs. 2.1%, *P *= 0.017). Moreover, there are a large number of superficial nerves in the abdominal wall, and the longer the incision, the more superficial nerve damage, and the more severe the postoperative pain. Excess length of incision will increase postoperative inflammatory reaction, and also limit the patient's respiration and movement, thus increasing postoperative complications such as pneumonia, bedsore, and thrombosis, and prolonging the length of hospital stay. Lu et al. reported that the time to first flatus (2.50 ± 0.79 vs.2.86 ± 0.76, *P *= 0.022), time to liquid diet (3.62 ± 0.64 vs. 4.20 ± 0.76 day, *P *< 0.001), and the need for analgesics (22% vs. 48%, *P *= 0.006) were significantly lower for patients who underwent NOSES (8). Consistent with this study, our study also revealed that the time to recovery of gastrointestinal function of patients in the NOSES group was significantly earlier compared with the LAP group (2.6 ± 0.8 vs. 3.6 ± 0.9 day, *P *= 0.037). Moreover, patients in the NOSES group suffered significantly less pain than those in the LAP group on day 1–3, while fewer patients required analgesia (12.5% vs. 33.3%, *P *< 0.001).

Another possible doubt of NOSES is the potential risk of cancer cell exfoliation and implantation. In present study, 5 (3.5%) patients developed local recurrences in the NOSES group and 6 (4.2%) patients developed local recurrence in the LAP group. The NOSES group had similar OS (*P* = 0.850) and DFS (*P* = 0.494) compared with the LAP group. Tang et al. reported on 186 patients who underwent total laparoscopic anterior resection with transrectal specimen extraction, and they found no difference in 5-year OS and DFS between NOSES and conventional laparoscopic surgery (15). Similarly, Liu and his colleague conducted a case control study comparing the clinical outcomes of 50 patients who underwent transrectal NOSES with same number of patients who underwent a conventional laparoscopically assisted approach. They suggested there were no differences in local recurrence rate (6% vs. 5%, *P *= 0.670), 3-yers DFS (86.7% vs. 88.0%, *P *= 0.945), and 3-years OS (95.6% vs. 96.0%, *P *= 0.708) (8). The above literatures are basically consistent with our results. We believe that the NOSES technology has no additional technical difficulties in following the oncological principles, especially during high ligation of inferior mesenteric artery and bowel mobilization. In the process of specimens extraction, strictly follow the principle of colorectal cancer-free, which will not increase the possibility of tumor exfoliation and implantation.

The present study have several limitations. Firstly, the operations of 288 patients were performed by 5 different surgeons, which would lead to a certain technical imbalance. However, these 5 experts have at least 500 cases of laparoscopic experience of CRC treatment and completed the learning curve for NOSES before data collection. Secondly, this study is a retrospective study and there must have inherent selection bias. However, we conducted a case-matched design to minimize selection bias.

## Conclusion

In conclusion, the transrectal NOSES procedure is a well-established strategy with advantages in reducing postoperative pain, faster recovery of gastrointestinal function, and less incision-related complications. In addition, the long-term survival is similar between NOSES and conventional laparoscopic surgery.

## Data Availability

The original contributions presented in the study are included in the article/Supplementary Material, further inquiries can be directed to the corresponding authors.
